# PPARγ: a key orchestrator of epidermal barrier, immune responses, and lipid metabolism in atopic dermatitis pathogenesis and therapy

**DOI:** 10.3389/falgy.2026.1780908

**Published:** 2026-03-13

**Authors:** Yuting Bao, Kexin Xu, Yue Du, Meng Feng, Li Li, Liangchang Li, Guanghai Yan, Xiaowan Li

**Affiliations:** Department of Medical College, Yanbian University, Yanji, China

**Keywords:** agonists, atopic dermatitis, PPARγ, skin barrier function, targeted therapy

## Abstract

Atopic dermatitis (AD) is an immune-mediated inflammatory dermatosis characterized by epidermal barrier dysfunction, immune dysregulation, and cutaneous microbial dysbiosis. Existing therapeutic modalities for AD are limited in efficacy and durability, highlighting an unmet clinical need for novel, safe, and effective treatment strategies. Peroxisome proliferator-activated receptor gamma (PPARγ), a pivotal nuclear receptor involved in metabolic and inflammatory regulation, has emerged as a promising therapeutic target for AD. Its pleiotropic mechanisms encompass the restoration of stratum corneum integrity, modulation of aberrant immunoinflammatory signaling, normalization of cutaneous lipid metabolism, and regulation of the cutaneous microbiome and neuroimmune circuitry. This review comprehensively synthesizes the mechanistic evidence linking PPARγ to AD pathogenesis and critically appraises its potential as a novel therapeutic.

## 1 Introduction

AD is a globally prevalent and profoundly burdensome inflammatory dermatosis, with an estimated worldwide prevalence of approximately 1.5% and incidence has shown an alarming upward trajectory in recent years (1, 2). Current clinical management of AD remains a formidable challenge. Due to its complex, multi-factorial etiopathogenesis and a highly heterogeneous clinical course. The disease frequently manifesting as chronic relapsing or refractory flares that severely compromise patients’ quality of life (3, 4). Consequently, advancing the mechanistic understanding of AD pathogenesis and identifying novel, efficacious therapeutic targets are critical and unmet priorities in dermatological research.

PPARγ, a lipid-sensing transcription factor, regulates genes involved in lipid metabolism, adipogenesis, and inflammation (5). It facilitates epidermal barrier repair, modulates Th1/Th2/Th17 immune imbalance—which attenuates IgE production—and helps maintain cutaneous microbial homeostasis (6, 7). The central role of PPARγ in barrier restoration, immune modulation, and metabolic homeostasis is thus highly consistent with the core pathophysiological features of AD: barrier dysfunction, immune dysregulation, and inflammatory amplification (8, 9).

Consequently, PPARγ is a compelling therapeutic target for the management of AD. This review endeavors to elucidate the intricate regulatory mechanisms of PPARγ in AD pathogenesis and to underscore its clinical relevance, providing a theoretical foundation for developing PPARγ-targeted therapies for AD and other inflammatory diseases.

## 2 Structure and function of PPARγ

### 2.1 Gene structure and protein domain composition of PPARγ

As a key member of the nuclear receptor superfamily, PPARγ possesses a gene architecture and protein domain organization that are central to regulating diverse biological pathways. Its transcription is modulated by distinct promoters and alternative 5’ exons, yielding three major mRNA variants which encode the isoforms PPARγ1, PPARγ2, and PPARγ3 (10). Structurally, the gene encompasses regulatory elements, coding sequences, and polymorphic loci. The regulatory regions orchestrate PPARγ transcriptional activity by binding endogenous ligands and exogenous signaling molecules, while the coding sequence undergoes precise exon-intron splicing to generate its critical functional domains (11). Notably, genetic variations in the PPARγ locus, particularly common single-nucleotide polymorphisms (SNPs), are strongly linked to an increased susceptibility to major metabolic disorders, including diabetes, cardiovascular diseases, and obesity (12).

**Figure 1:**
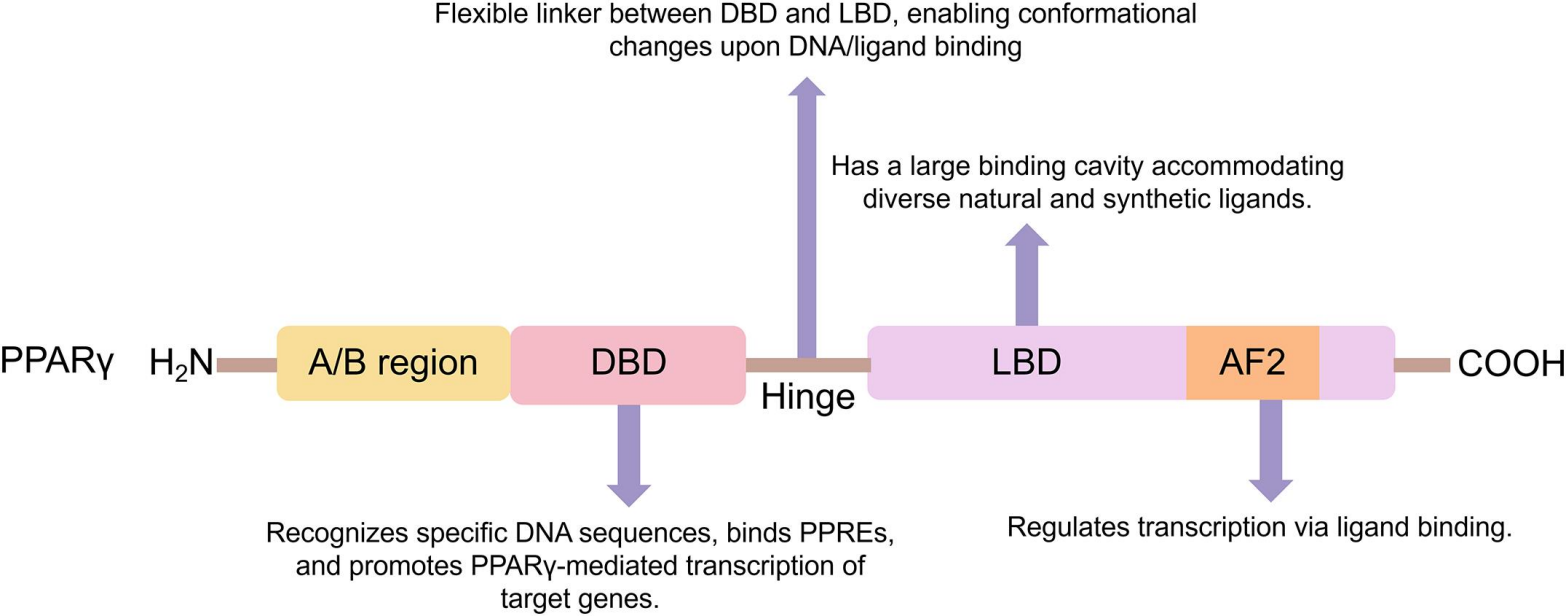
Schematic of PPARγ functional domain architecture. This illustration depicts PPARγ's key functional domains (A/B, DBD, flexible hinge, LBD/AF2), which collaboratively mediate target gene transcriptional regulation through DNA recognition and ligand engagement.

The PPARγ protein structure is characterized by distinct functional domains, each contributing a specific role to the sequential execution of its transcriptional activities (13). A/B Domain (AF-1):Situated at the N-terminus, this domain facilitates ligand-independent transcriptional activation and serves as a crucial interface for interactions with other transcription factors and coregulators. Its transcriptional activity is precisely modulated by various post-translational modifications, with phosphorylation representing a significant regulatory mechanism. C Domain (DNA-Binding Domain, DBD):Located centrally, the DBD is responsible for the sequence-specific recognition of and binding to peroxisome proliferator response elements (PPREs) within the regulatory regions of target genes, thereby initiating the gene program regulated by PPARγ. D Domain (Hinge Region): This region acts as a flexible linker between the DBD and the ligand-binding domain (LBD), imparting the conformational flexibility necessary for accommodating structural shifts induced by DNA binding or ligand engagement (14). E/F Domain (Ligand-Binding Domain, LBD): Found at the C-terminus, the LBD encompasses the ligand-dependent activation function 2 (AF-2), and its conformational rearrangement upon ligand binding directly determines transcriptional output (15). The LBD possesses a capacious hydrophobic ligand-binding pocket that accommodates a wide array of endogenous ligands (e.g., fatty acids, 15-deoxy-Δ^12,14^-prostaglandin J_2_) and synthetic agonists such as thiazolidinediones (TZDs). Ligand occupancy promotes the heterodimerization of PPARγ with the retinoid X receptor (RXR), which facilitates the recruitment of transcriptional coactivator complexes and subsequently drives the activation of downstream target gene expression (16) (Figure 1).

### 2.2 PPARγ ligands

PPARγ ligands can be broadly classified into synthetic and endogenous/natural subsets (17). Among synthetic agonists, thiazolidinediones (TZDs), typified by rosiglitazone and pioglitazone, represent classic examples (18). Although synthetic ligands have been extensively investigated, natural ligands offer distinct advantages in terms of greater accessibility and an improved safety profile. Notably, plant-derived natural ligands—including himbacines, genistein, chrysin, and honokiol—have emerged as promising candidates (19). Collectively, these findings provide robust evidence for the therapeutic potential of PPARγ modulators in treating inflammatory skin conditions and lay a solid theoretical foundation for their expanded application in inflammatory disease pathogenesis (20, 21).

### 2.3 Tissue distribution and cutaneous expression of PPARγ

PPARγ exhibits tissue-specific distribution, with its expression and function varying across organs. While it plays key roles in lipid metabolism and glucose homeostasis in tissues such as adipose, colon, and spleen, its expression in the skin is of direct relevance to AD pathogenesis (22–33).

**Figure 2:**
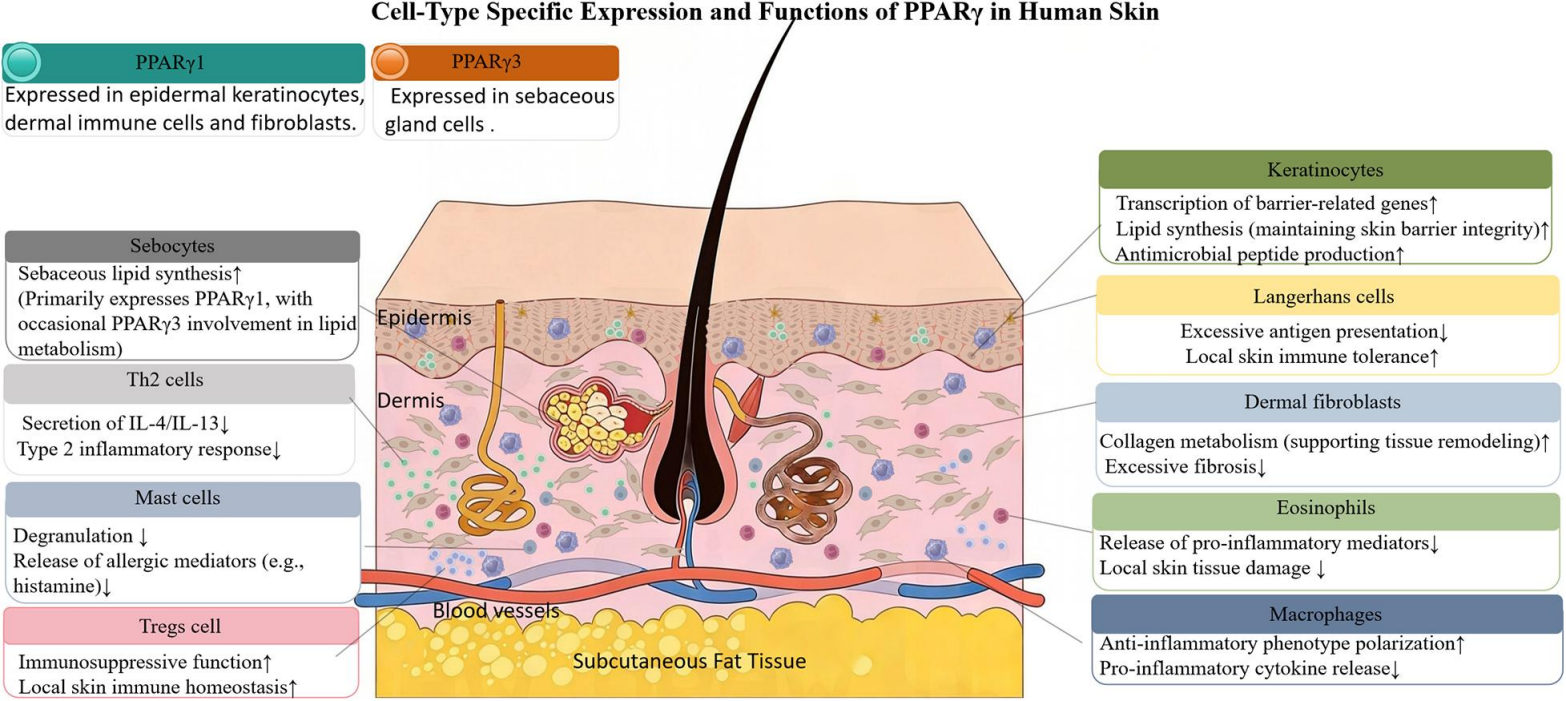
Expression and functional specificity of PPARγ in human skin cells. Layer-specific PPARγ localization is depicted in epidermal keratinocytes, dermal immune cells/fibroblasts, and sebaceous gland cells, reflecting distinct functional roles.

In the skin, PPARγ expression exhibits marked cell-type specificity, undergoing distinct alterations under various pathological conditions and showing selective distribution of its isoforms (34). Keratinocytes express PPARγ in their nuclei across all epidermal layers, with particularly abundant expression in the granular and basal strata. In these cells, PPARγ promotes the transcription of barrier-related genes, regulates lipid biosynthesis to maintain skin barrier integrity, and enhances the production of antimicrobial peptides (35). Within cutaneous immune compartments, PPARγ drives the polarization of anti-inflammatory macrophages, strengthens the suppressive function of regulatory T cells, inhibits mast cell degranulation and eosinophil-mediated tissue damage, and rectifies localized immune dysregulation (36). In dermal fibroblasts, PPARγ modulates collagen metabolism to support tissue remodeling while preventing excessive fibrosis. In sebocytes, it governs sebum lipid synthesis; dysregulated PPARγ expression under inflammatory or metabolic stress contributes to abnormal sebum secretion (37). Regarding isoform distribution, PPARγ1 is the predominant isoform expressed in epidermal keratinocytes, dermal immune cells, and fibroblasts, where it orchestrates skin barrier repair and immunomodulation. PPARγ2 is virtually absent in human skin and is not considered functionally relevant in this organ. PPARγ3 is occasionally identified in sebocytes and is hypothesized to participate in the regulation of lipid metabolism in sebaceous glands (38) (Figure 2).

## 3 Role of PPARγ in the pathogenesis of atopic dermatitis

The pathogenesis of AD is initiated by a compromised epidermal barrier, which precipitates a dysregulated inflammatory cascade. This primary defect is intricately linked to concomitant lipidomic dysregulation and cutaneous dysbiosis, which collectively drive the development of chronic inflammatory dermatoses (39). The cardinal pathological features of AD—including aberrant barrier protein expression, immune dysregulation, altered lipid profiles, and overcolonization by *Staphylococcus aureus*—synergistically promote epidermal neuro-hypersensitivity. This, in turn, fuels a vicious, self-perpetuating itch-scratch cycle, establishing a pathogenic feedback loop that sustains chronic inflammation and barrier dysfunction (40).

### 3.1 PPARγ and the skin barrier in AD

A compromised skin barrier underlies AD, with a critical deficiency in the nuclear receptor PPARγ driving epidermal pathology (41). Downregulation of PPARγ in lesional AD skin cripples its transcriptional regulation of key structural proteins (42). Restoring PPARγ activity directly addresses this deficit by binding PPAR response elements (PPREs) in target gene promoters, potently upregulating filaggrin (FLG) and loricrin (LOR) to centralize barrier repair (43, 44).

Furthermore, PPARγ activation enhances epidermal barrier function through the regulation of ceramide synthesis enzymes such as serine palmitoyltransferase and ceramide synthases, facilitating proper epidermal lipid lamellae formation (45).

Filaggrin (FLG)deficiency, a key AD hallmark, arises from genetic mutations or transcriptional suppression, fundamentally disrupting profilaggrin processing into functional monomers (46). This abrogates keratin filament aggregation, compromising corneocyte cohesion and barrier integrity. Impaired FLG degradation further curtails natural moisturizing factor (NMF) production, driving transepidermal water loss (TEWL) and escalating xerosis (47). PPARγ activation rescues FLG expression and processing, thereby fortifying the stratum corneum and restoring cutaneous hydration (48, 49).

**Figure 3:**
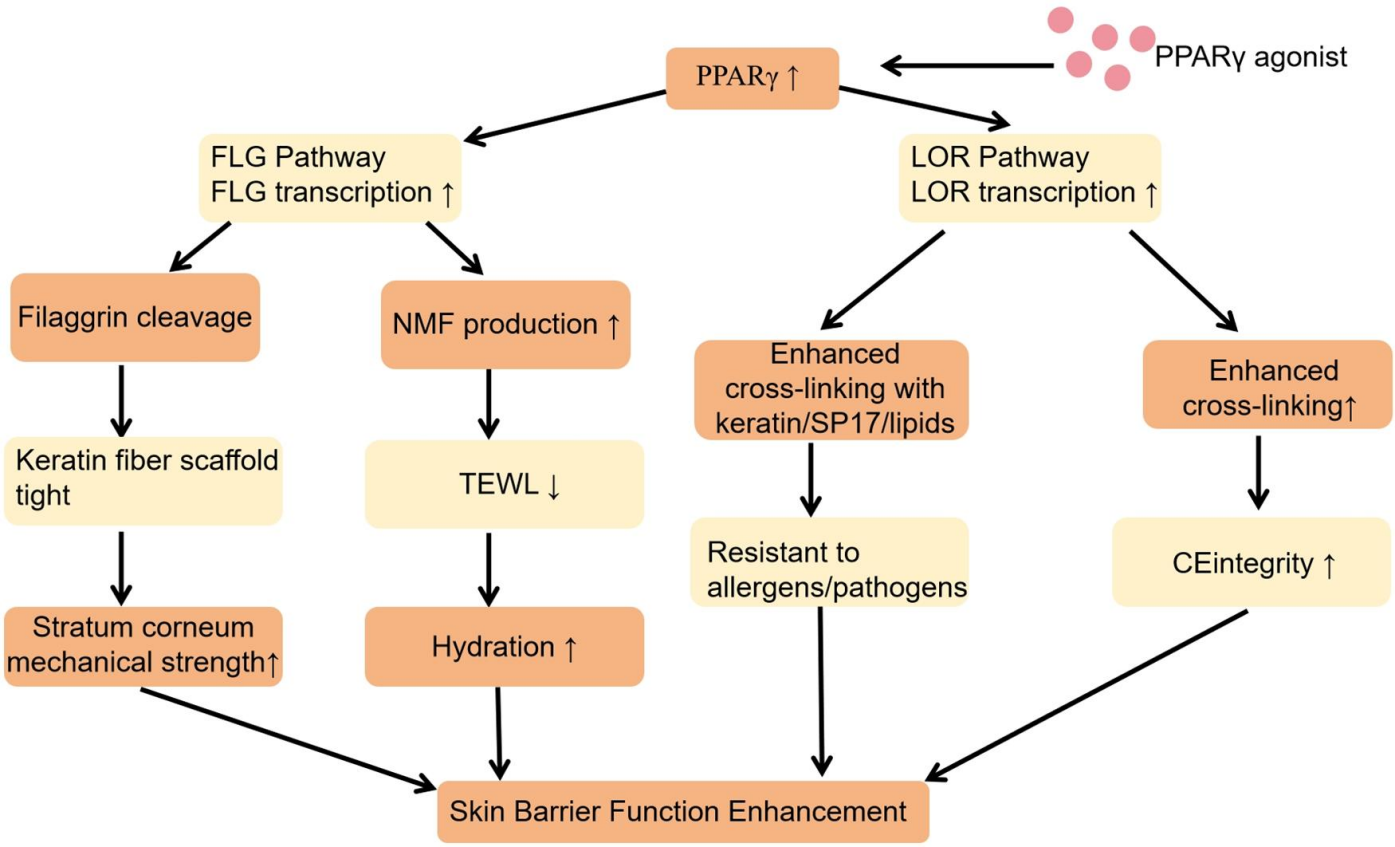
PPARγ downregulation suppresses filaggrin and loricrin pathways to induce skin barrier impairment in atopic dermatitis. Reduced PPARγ expression leads to skin barrier impairment via disruption of FLG and LOR pathways, as illustrated here. This mechanism represents a therapeutic target for PPARγ agonists.

Loricrin (LOR), another key PPARγ target, is indispensable for cornified envelope (CE) formation—the cornerstone of corneocyte structural integrity (50). Deficient LOR in AD compromises CE assembly, diminishing epidermal mechanical and chemical resilience and abrogating its barrier function against xenobiotic entry (51). Upregulation of LOR by PPARγ facilitates its cross-linking with keratin and lipids, thereby restoring robust CE structure and bolstering the skin's defense against pro-inflammatory insult (52) (Figure 3).

### 3.2 PPARγ and the inflammatory response in AD

The inflammatory cascade in AD unfolds in distinct acute and chronic phases (53). The acute inflammatory response is canonically driven by IgE-mediated type I hypersensitivity, underpinned by a dominant Th2 cytokine milieu (54). During this phase, IL-4 and IL-13, predominantly secreted by activated Th2 cells, type 2 innate lymphoid cells (ILC2s), and mast cells orchestrate B-cell differentiation, leading to the robust synthesis and systemic release of IgE (55). Subsequent allergen exposure triggers cross-linking of IgE bound to FcεRI receptors on mast cells and basophils (56). This event precipitates cellular degranulation and the release of inflammatory mediators, such as histamine, which manifest as the characteristic acute symptoms of AD (57). Critically, activated of PPARγ in antigen-presenting cells (e.g., dendritic cells) and T lymphocytes directly targets this pathogenic axis by downregulating IL-4 and IL-13 gene expression (58). This suppression curtails IgE overproduction and attenuates FcεRI-mediated effector cell activation, thereby halting the cascade at its source (59).

**Figure 4:**
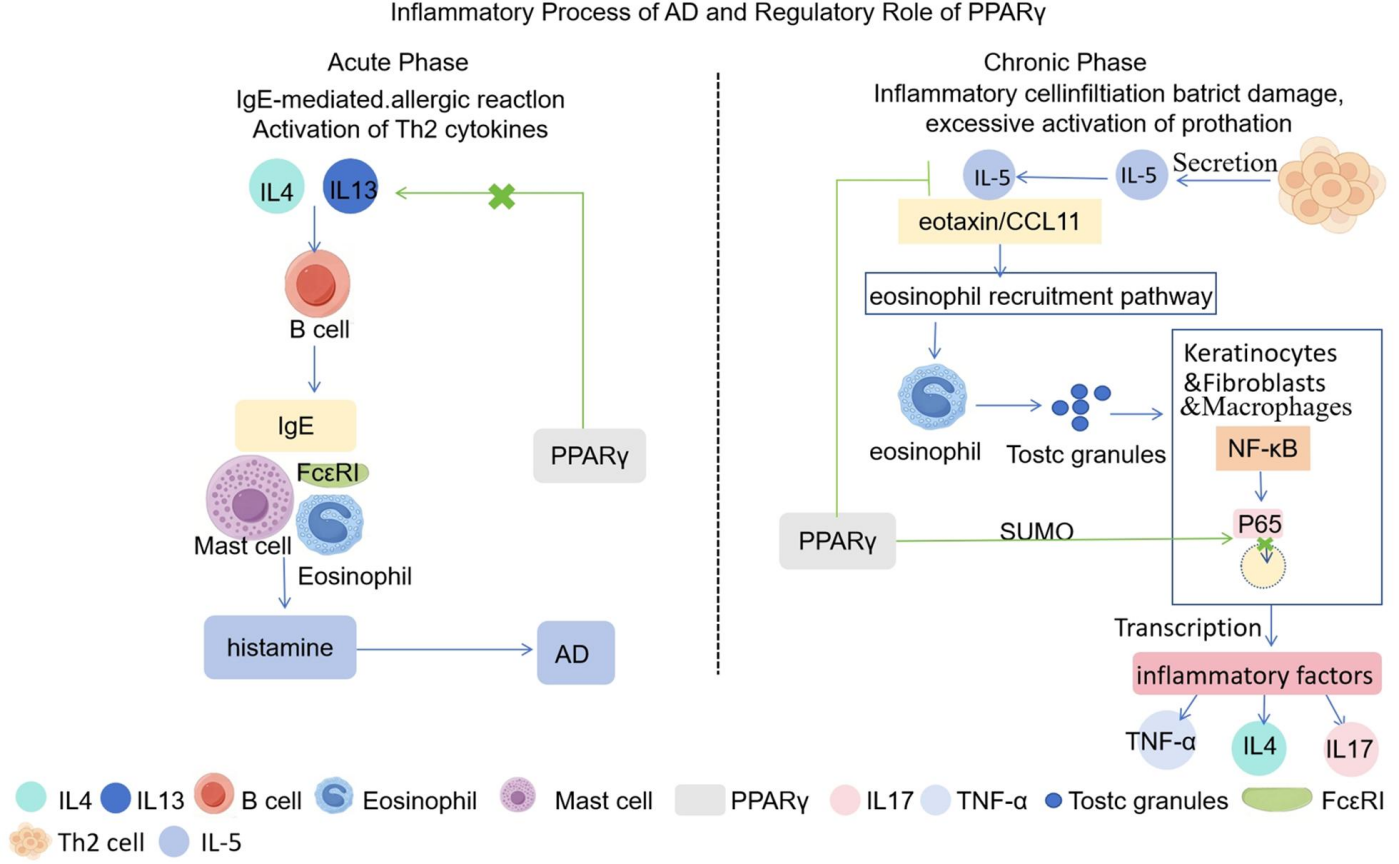
PPARγ regulates pathogenic inflammatory cascades across the acute and chronic phases of atopic dermatitis. This illustration depicts PPARγ regulating AD's inflammatory phases: blocking IL-4/IL-13 to inhibit acute-phase IgE reactions, and suppressing IL-5/p65 activity to mitigate chronic-phase inflammation.

The transition to the chronic phase of AD is characterized by persistent inflammatory cell infiltration, progressive epidermal barrier dysfunction, and robust activation of pro-inflammatory pathways (60). Specifically, IL-5, primarily derived from Th2 cells, works synergistically with eotaxin (CCL11) to drive eosinophil recruitment and activation (61). These activated eosinophils liberate cytotoxic proteins, perpetuating tissue damage and contributing to the activation of the NF-κB signaling pathway in keratinocytes, dermal fibroblasts, and infiltrating immune cells (62, 63). Nuclear translocation of the p65 subunit then ensues, driving the transcription of potent pro-inflammatory cytokines, including IL-4, IL-17, and TNF-α. PPARγ activation resolves this protracted inflammation through a dual mechanism: it suppresses IL-5 expression in Th2 cells and via SUMOylation of the p65 subunit, inhibits its nuclear translocation specifically in macrophages and keratinocytes (27, 64). Additionally, PPARγ modulates the function of dendritic cells (DCs) and Langerhans cells (LCs), key antigen-presenting cells in AD pathogenesis, by reducing their maturation and pro-inflammatory cytokine production, thereby attenuating T cell activation (65) (Figure 4).

### 3.3 PPARγ and lipid metabolism in AD

Patients with AD display a profound dysregulation of cutaneous lipid metabolism, characterized by an aberrant composition of the epidermal stratum corneum lipids and a compromised antimicrobial defense (66). The nuclear receptor PPARγ serves as a central regulator of skin lipid homeostasis in a cell-type-specific manner. In keratinocytes, which are the primary site for epidermal lipid synthesis, PPARγ orchestrates this process, in part, by modulating the expression of key effector molecules such as CD36, thereby coordinating epidermal fatty acid transport, the synthesis of ceramides and unsaturated fatty acids, and the subsequent repair of the skin barrier (67, 68).

In lesional AD skin, diminished CD36 expression in keratinocytes impairs fatty acid uptake, directly contributing to lipid barrier dysfunction (69, 70). Agonist-activated PPARγ counteracts this deficit in keratinocytes by forming a heterodimer with the retinoid X receptor (RXR). This complex binds to specific peroxisome proliferator response elements (PPREs) in the promoter region of the CD36 gene, driving its transcription (71). The resulting restoration of CD36-mediated fatty acid import provides critical metabolic substrates necessary for barrier reconstruction (72).

The fatty acids palmitate and stearate, whose cellular uptake is facilitated by CD36, are essential precursors for denovo ceramide synthesis—a pathway notably deficient in AD epidermis. By upregulating CD36, PPARγ activation replenishes this precursor pool. Moreover, PPARγ may directly enhance ceramide production by regulating key enzymes in the sphingolipid biosynthesis pathway, such as serine palmitoyltransferase and ceramide synthases, thereby fortifying the lipid barrier (73, 74).

Beyond ceramide metabolism, PPARγ activation in keratinocytes and sebocytes induces the expression of enzymes responsible for fatty acid desaturation and elongation, notably stearoyl-CoA desaturase 1 (SCD1) and members of the elongation of very long-chain fatty acids (ELOVL) family (45). This shifts the epidermal lipid profile from saturated toward unsaturated species, generating long-chain polyunsaturated fatty acids including eicosapentaenoic acid (EPA) and docosahexaenoic acid (DHA). These lipids serve a dual function: they optimize the physical properties of the stratum corneum to enhance water retention and, through their inherent anti-inflammatory actions, synergize with PPARγ to suppress the release of pro-inflammatory cytokines (75, 76). Moreover, PPARγ activation in skin-resident macrophages and dendritic cells can modulate their lipid metabolism and inflammatory phenotype, contributing to the resolution of inflammation (77).

### 3.4 PPARγ and the skin microecology in AD

PPARγ, a lipid-sensitive nuclear transcription factor, is essential for sustaining skin barrier integrity and immune homeostasis. Its involvement in AD pathogenesis extends to the modulation of the skin microbiome (78). Patients with AD frequently exhibit insufficient secretion of antimicrobial peptides (AMPs) (79), including human beta-defensins (HBD-2, HBD-3) and cathelicidin, leading to the over-colonization of *Staphylococcus aureus* (50, 80). Upon activation, PPARγ upregulates the expression of these AMPs, directly inhibiting *S. aureus* biofilm formation and proliferation (81). Concurrently, PPARγ activation mitigates pathogen-induced inflammatory responses by reducing the release of pro-inflammatory cytokines and inducing M2 anti-inflammatory macrophage polarization, thereby preventing further skin barrier deterioration (82). Crucially, PPARγ-regulated AMPs possess selective antimicrobial properties, enabling targeted clearance of *S. aureus* while preserving commensal flora, such as Staphylococcus epidermidis (83). However, direct evidence linking PPARγ activation to species-specific microbiome modulation remains an area for further investigation. Moreover, these AMPs synergize with bacteriocins, like lugdunin, produced by commensals to further suppress pathogenic bacterial colonization (84). While plausible, this proposed synergy requires additional experimental validation within the context of PPARγ signaling. Ultimately, this coordinated action by PPARγ reshapes the skin microbiome equilibrium, providing a robust foundation for skin health (85).

### 3.5 PPARγ and the neuroimmune circuit of AD

The neuroimmune pathogenesis of AD exhibits distinct manifestations in the central and peripheral nervous systems. At the central nervous system (CNS) level, this is characterized by heightened excitability of spinal dorsal horn neurons, amplification of itch signaling mediated by neurotransmitters such as neurokinin B (NKB) and gastrin-releasing peptide (GRP), and central sensitization (86). Centrally, PPARγ orchestrates a potent counter-regulatory response. Activation of the PPARγ/nuclear factor-kappa B (NF-κB) pathway suppresses microglial activation, thereby mitigating CNS inflammation-induced neuronal hyperexcitability. Concurrently, it indirectly reduces the release of neurotransmitters from peripheral afferents projecting to the spinal cord, obstructing the central amplification of itch signals and reversing central sensitization (87).

In contrast, the peripheral nervous system (PNS) in AD is primarily defined by aberrant epidermal nerve fiber (IENF) proliferation, enhanced sensitivity of sensory neurons to pruritogens, and a vicious itch-scratch cycle driven by neurogenic inflammation (88, 89). At the peripheral level, PPARγ counteracts these pathological processes. Through the upregulation of tyrosine protein phosphatase (SHP-2), it inhibits the Rho/ROCK pathway, thereby curbing the excessive sprouting and increased density of IENFs. Furthermore, PPARγ downregulates the expression and function of sensory neuron ion channels, such as transient receptor potential vanilloid 1 (TRPV1) and transient receptor potential ankyrin 1 (TRPA1), thus impeding the binding of pruritogens to their receptors (90). This dual action not only reduces nerve fiber exposure but also promotes keratinocyte differentiation and epidermal barrier repair, ultimately ameliorating the deleterious cycle of “barrier damage - neuronal activation - inflammation – itch” (91, 92).

## 4 Application of PPARγ agonists in AD treatment

### 4.1 Synthetic PPARγ agonists

Thiazolidinediones (TZDs), including rosiglitazone and pioglitazone, are synthetic agonists of PPARγ. They share the ability to suppress inflammation in epithelial and immune cells, reduce pro-inflammatory cytokines, and restore skin barrier integrity by modulating keratinocyte function and lipid metabolism (93). Rosiglitazone primarily reduces inflammation via PPARγ activation. Pioglitazone further enhances keratinocyte proliferation/differentiation and lipid synthesis, alleviating dryness and scaling (94). PPARγ/α dual agonists, classified into first- and next-generation agents, aim for synergistic inflammation and cutaneous metabolism modulation (95). First-generation dual agonists showed preclinical anti-inflammatory and skin barrier benefits but faced clinical safety hurdles (96). Saroglitazar, a novel dual agonist, retains potent anti-inflammatory efficacy without severe adverse reactions. Currently investigated for AD, it represents a promising direction for synthetic PPAR agonist development (97).

### 4.2 Natural PPARγ agonists

Natural agonists of PPARγ can be broadly classified into endogenous and exogenous compounds. Representative endogenous agonists include 15-deoxy-Δ^12,14^-prostaglandin J_2_ (15d-PGJ_2_) and omega-3 fatty acids, such as docosahexaenoic acid (DHA) and eicosapentaenoic acid (EPA) (98). Specifically, 15d-PGJ_2_ activates PPARγ to suppress cutaneous inflammation and promote keratinocyte differentiation, thereby ameliorating skin barrier structure and function (99). Omega-3 fatty acids, in addition to attenuating inflammatory cascades via PPARγ activation, rebalance dermal lipid metabolism and enhance skin barrier stability, consequently mitigating dermatological conditions associated with barrier dysfunction. Exemplary exogenous PPARγ agonists encompass extracts from Cistanche tubulosa and curcumin (100, 101). These agents activate the PPARγ signaling pathway, eliciting pleiotropic biological effects. Firstly, they exhibit potent antioxidant properties by scavenging reactive oxygen species (ROS), thereby reducing oxidative stress-induced damage to skin tissues. Secondly, they inhibit inflammatory responses through decreased release of pro-inflammatory cytokines and concurrently protect the skin barrier, collectively preserving a stable cutaneous microenvironment. These findings establish a robust theoretical foundation for the therapeutic application of natural active ingredients in inflammatory dermatoses (102) (Table 1).

**Table 1:** Classification, mechanisms of action, and atopic dermatitis-related therapeutic efficacy of PPARγ agonists.

Agonist Category	Specific Representatives	Mechanism of Action	AD Treatment-related Efficacy
Synthetic agonists	Rosiglitazone (thiazolidinediones)	Directly activates PPARγ; inhibits the release of pro-inflammatory factors such as TNF-α and IL-4 in skin tissue (84)	Alleviates inflammatory manifestations like erythema and pruritus (84)
Synthetic agonists	Pioglitazone (thiazolidinediones)	Activates PPARγ plus weak PPARα agonistic activity; inhibits inflammatory infiltration; promotes keratinocyte proliferation and barrier repair (85)	Improves skin dryness and desquamation; exerts both anti-inflammatory effect and barrier repair (85)
Synthetic agonists	Early glitazars (PPARγ/α dual agonists)	Dual activation of PPARγ/α; synergistically regulates inflammation and skin metabolism (86)	Exhibits significant anti-inflammatory and barrier repair effects in animal experiments (87)
Synthetic agonists	Saroglitazar (novel PPARγ/α dual agonists)	Dual activation of PPARγ/α; maintains excellent anti-inflammatory activity (88)	Remarkable anti-inflammatory efficacy without severe side effects (88)
Natural agonists	Plant-derived (Michelia lactone, Genistein, Chrysin, Honokiol)	Regulates PPARγ signaling pathway; up-regulates PPARγ expression; induces M2 polarization of macrophages (89)	Inhibits skin inflammatory response; alleviates oxidative stress damage; promotes skin barrier repair
Natural agonists	Plant-derived (Myristicin lignan)	Dual activation of PPARγ/α; synergistically regulates skin inflammatory microenvironment and lipid metabolism disorders (90)	Achieves both anti-inflammatory effect and metabolic regulation (91)
Natural agonists	Animal-derived (Camel milk)	Dual activation of PPARα/γ; inhibits inflammatory cell infiltration and tissue edema in skin (92)	Relieves symptoms of acute AD exacerbation (92)

### 4.3 Status of basic experiments and clinical research

Preclinical studies have demonstrated that PPARγ activation effectively suppresses inflammatory cytokines and promotes skin barrier repair, while avoiding the typical adverse effects associated with conventional corticosteroid therapies (83). In clinical practice, the efficacy and safety profile of PPARγ agonists for AD are critically dependent on the route of administration (103).Topical application allows for targeted modulation of lesional skin, effectively reducing erythema and pruritus, and decreasing *Staphylococcus aureus* colonization (104). Combination therapy with topical calcineurin inhibitors yields synergistic effects. Owing to minimal systemic exposure, topical PPARγ agonists are suitable for long-term maintenance therapy in patients aged two years and older, particularly for mild-to-moderate AD involving sensitive skin areas (105).

In contrast, systemic administration provides rapid control in patients with extensive lesions and systemic inflammation, representing a viable option for moderate-to-severe AD refractory to topical treatments. However, long-term monotherapy is often limited by tachyphylaxis, and oral formulations are associated with a higher incidence of adverse events (106). Caution is advised in elderly patients and those with cardiovascular comorbidities, while use is contraindicated during pregnancy, lactation, and in patients with severe hepatic or renal impairment.

Although PPARγ modulation is well-established in the management of metabolic disorders, its clinical translation in dermatology and neurology has been hindered by a lack of pharmacological selectivity. Future development should focus on the design of novel, highly selective PPARγ agonists and their evaluation in well-designed Phase II combination trials (107, 108).

#### 4.3.1 Current limitations and translational challenges

Current evidence supporting PPARγ agonists for AD treatment remains primarily preclinical, with most data derived from *in vitro* keratinocyte studies and mouse models (109). While early topical agonist trials show promise, several important limitations must be acknowledged (110). Systemic PPARγ agonists carry established safety risks including weight gain, fluid retention, and cardiovascular effects that may limit their long-term use in dermatology (111). The lack of tissue-selective PPARγ modulators presents a significant pharmacological challenge. Furthermore, key clinical questions regarding optimal dosing regimens, long-term efficacy across diverse AD phenotypes, and comparative effectiveness vs. existing therapies remain unanswered. These limitations highlight the need for careful consideration of risks vs. benefits and the importance of developing targeted PPARγ modulators specifically optimized for dermatological applications (112).

## 5 Discussion

**Figure 5:**
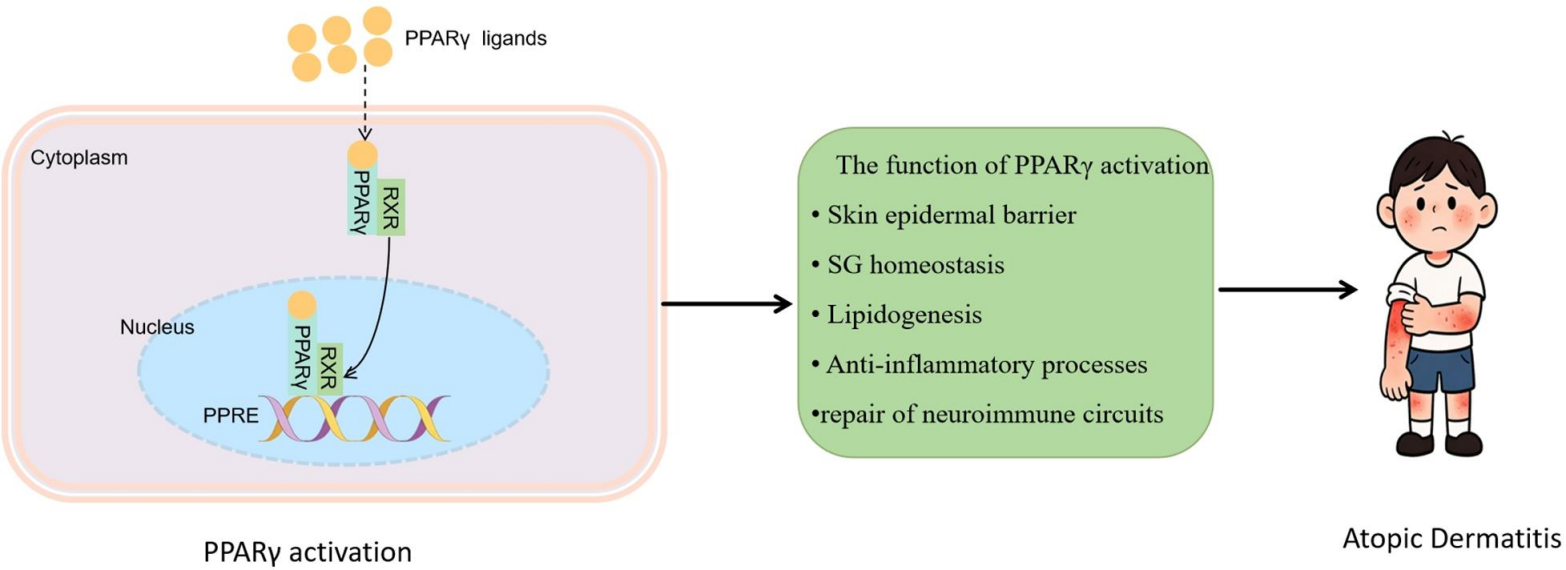
PPARγ ligand-induced PPARγ/RXR heterodimerization and PPRE binding mediates multifaceted therapeutic effects in atopic dermatitis. This illustration depicts that PPARγ ligands drive PPARγ to form a dimer with RXR, which binds PPRE; this activates functions to target atopic dermatitis.

PPARγ ligands hold considerable therapeutic promise for AD due to their multifaceted mechanism of action, which includes robust skin barrier restoration, pronounced anti-inflammatory activity, enhanced lipid metabolism, and regulation of the skin microbiome (67). Crucially, in AD patients coinfected with metabolic comorbidities like obesity, the dual metabolic and anti-inflammatory properties of PPARγ ligands present significant therapeutic benefits. They can concurrently alleviate cutaneous inflammation and metabolic dysregulation, fostering improved patient adherence and enabling personalized treatment strategies for complex AD phenotypes (113) (Figure 5).

However, it is important to note that the effects of PPARγ activation are not uniformly beneficial and can be context-dependent. For instance, while PPARγ activation generally promotes barrier repair, its sustained stimulation in sebocytes under certain conditions may exacerbate lipogenesis, potentially worsening sebum dysregulation in acne-prone or seborrheic skin—a relevant consideration for AD patients with concurrent sebaceous disorders (114). Furthermore, the pharmacological profile of the ligand critically influences outcomes: full agonists (e.g., TZDs) may induce excessive adipogenesis and systemic metabolic side effects, whereas partial agonists or selective PPARγ modulators (SPPARγMs) appear to retain anti-inflammatory efficacy while mitigating adverse metabolic consequences (115, 116). These differential effects underscore the necessity for ligand-specific therapeutic strategies in dermatology. Additionally, some studies suggest that PPARγ signaling may have cell-type–specific or even paradoxical effects; for example, while it generally suppresses inflammation, in certain immune contexts it might inadvertently support pro-fibrotic pathways in dermal fibroblasts under chronic inflammatory conditions (23). This complexity highlights that PPARγ's role is not uniformly positive and requires careful modulation based on disease context, cell type, and ligand class.

Critically, substantial knowledge gaps and translational challenges persist. First, the complex crosstalk between PPARγ ligands and pivotal signaling pathways, such as NF-κB, Wnt, and Nrf2, remains incompletely understood, and their context-dependent regulatory networks require further systematic elucidation (117). Second, the bioactivity and consistency of natural ligands are susceptible to variability in plant cultivation conditions and extraction protocols, while carrier preparation may introduce bioactive or cytotoxic contaminants. Moreover, existing delivery systems necessitate further optimization in encapsulation efficiency, controlled release kinetics, and long-term biocompatibility (118). Perhaps most importantly, the currently available clinical evidence is largely constrained by study design limitations—notably modest cohort sizes and relatively short follow-up durations—reflecting the absence of a robust, staged translational framework linking preclinical mechanistic insights to clinical outcomes (119). Consequently, establishing definitive causal relationships between fundamental molecular findings and demonstrable therapeutic efficacy represents an urgent and unmet translational priority.

Advancing PPARγ-targeted therapy for AD necessitates a dual approach: the development of highly selective ligands to reduce off-target sequelae and the design of innovative, biocompatible delivery platforms (e.g., microbial carriers, smart formulations) for improved target engagement: PPARγ has emerged as one of the most extensively studied transcription factors since its discovery in 1990, highlighting its importance in the etiology and treatment of numerous diseases involving various types of cancer, type 2 diabetes mellitus, autoimmune, dermatological and cardiovascular disorders (120). Ligands are regarded as the key determinant for the tissue-specific activation of PPARγ. However, the mechanism governing this process is merely a contradictory debate which is yet to be systematically researched. Either these receptors get weakly activated by endogenous or natural ligands or leads to a direct over-activation process by synthetic ligands, serving as complete full agonists (121). Therefore, fine-tuning on the action of PPARγ and more subtle modulation can be a rewarding approach which might open new avenues for the treatment of several diseases. In the recent era, researchers have sought to develop safer partial PPARγ agonists in order to dodge the toxicity induced by full agonists, akin to a balanced activation (122). With a particular reference to cancer, this review concentrates on the therapeutic role of partial agonists, especially in cancer treatment (21). Additionally, a timely examination of their efficacy on various other disease-fate decisions has been also discussed. Mechanistic investigation must delve into the sophisticated interplay between PPARγ signaling and the skin-microbiome-gut axis. From a clinical perspective, robust, long-term studies are indispensable for validating ligand performance and safety across diverse AD phenotypes, including pediatric and obesity-driven presentations. Establishing robust manufacturing standards and regulatory pathways for combined ligand–delivery systems is a critical translational step. By fostering interdisciplinary collaboration, PPARγ-targeted interventions are poised to become a cornerstone of AD management, leveraging their pleiotropic effects to address the disease's complexity and enable the development of personalized, safe, and efficacious therapeutic strategies for patients (123, 124).
